# Antibacterial Activity of Polyaniline Coated in the Patterned Film Depending on the Surface Morphology and Acidic Dopant

**DOI:** 10.3390/nano12071085

**Published:** 2022-03-25

**Authors:** Shahkar Falak, Bo Kyoung Shin, Do Sung Huh

**Affiliations:** Department of Chemistry, Nano Science and Engineering, Center of Nano Manufacturing, Inje University, Gimhae-si 50834, Korea; sfalak1723@gmail.com (S.F.); sinbbo715@gmail.com (B.K.S.)

**Keywords:** polyaniline, micro-structured surface, sulfonated polyaniline, antibacterial, antibiofilm

## Abstract

We have fabricated poly(ε-caprolactone) (PCL) films with flat and honeycomb-patterned (HCP) structures to coat polyaniline (PANI) on the film surface. In addition, the effect of chemical modification of PANI by sulfuric acid (H_2_SO_4_) was also studied for antibacterial activity. The flat and HCP PCL films were obtained by simple evaporation of the solvent and via the breath figure (BF) method, respectively. The morphology and chemical composition of PANI coated on the film surface were evaluated by scanning electron microscopy (SEM) and X-ray spectroscopy (EDX). Fourier transform infrared spectroscopy (FT-IR) and thermogravimetric analyses (TGA) were obtained to identify the PANI coating. The wettability and conductivity of the films were also measured. Applicational aspects were evaluated by assessing antibacterial and antibiofilm activity against *Escherichia coli* (*E. coli*) and *Staphylococcus aureus* (*S. aureus*). The EDX, TGA, and FT-IR findings indicated chemical modification of PCL film by PANI and H_2_SO_4_. The conductivity of the films was increased by the coating of PANI to the patterned surface and additionally increased by the chemically modified PANI. The antibacterial activity was 69.79%, 78.27%, and 88% against *E. coli*, and 32.73%, 62.65%, and 87.97% against *S. aureus*, for flat PANI, HCP PANI, and H_2_SO_4_-treated HCP films, respectively. Likewise, the PANI coated flat, HCP, and H_2_SO_4_-treated HCP films inhibited *E. coli* biofilm formation by around 41.62%, 63%, and 83.88% and *S. aureus* biofilm formation by 17.81%, 69.83%, and 96.57%, respectively. The antibacterial activity of the HCP film was higher than that of flat PANI films, probably due to the higher coating of PANI on the HCP surface. Moreover, sulfonation of the HCP film with H_2_SO_4_ might have improved the wettability, thereby enhancing the antibacterial and antibiofilm properties. Our results showed that topographical changes, as well as doping, offer simple and cost-effective ways to modify the structural and functional properties of films.

## 1. Introduction

Polymers with specific properties are widely applied in biomedical fields such as tissue engineering and regenerative medicine [1]. Poly (ε-caprolactone) (PCL), poly (glycolic acid), poly (lactic acid), and poly (trimethylene carbonate) are mostly used due to their biodegradability. However, the contamination of polymeric materials with microorganisms, mostly bacteria, is still a major issue and causes nosocomial infections [2,3], as the long-term use of polymeric material suffers from microorganism fouling or biofilm formation [4]. Biofilms are coherent aggregates of bacterial cells embedded in a biopolymer matrix formed when cells attach to a solid substrate to survive [5]. The formation of biofilm makes the bacteria resistant, as it can persevere even in adverse environmental conditions, making it challenging to treat [6]. Therefore, to reduce the health complications caused by bacteria found on the polymeric material, interest in developing antibacterial polymer films or preventing the bacterial adhesion and biofilm formation on the surfaces has recently grown [7,8,9]. Fabrication of antifouling films alone may successfully delay or limit initial bacterial adhesion, but these films still cannot completely prevent bacterial growth on the film surface, which eventually leads to the formation of a biofilm [10]. Thus, developing novel membranes by introducing antibiofilm coatings to the bactericidal surfaces is a promising strategy to reduce biofilm formation [11].

Recently, researchers have applied various strategies to improve the antibacterial and antibiofilm properties, including the direct modification of the membrane material [12,13,14] or blending or coating with bioactive or chemical moieties having antibacterial properties such as metal ions or nanoparticles (NPs) [15,16] or quaternary ammonium compounds (QAC) [17]. Another approach is by improving hydrophilicity by utilizing hydrophilic polymers, such as poly (ethylene glycol) [18], polydopamine [19], zwitterions [20,21], or hydrophilic nanomaterials such as zinc oxide [22], and titanium oxide (TiO_2_) [23,24]. Some have even sought to modify the surface topography with micro/nanostructured features such as nanoneedles [25], spherical nanocones [26], nanopillars [27], nano spikes [28], etc. For example, Zhu et al. synthesized a poly (vinylidene fluoride) film containing QAC and zwitterion ions to study the antibacterial and antibiofilm activity of the films [2]. Although the modification of such films offers respectable antibacterial and antibiofilm activity due to its ability to kill or repel bacteria by contact, their fabrication is tedious, expensive, and requires highly skilled labor.

Polyaniline (PANI) is a well-known conducting polymer that is frequently investigated because of its attractive properties, namely, antibacterial properties, biocompatibility, and a simple acid–base doping–dedoping process [29,30,31]. PANI films can simply be modified through protonation with various dopant acids, thereby altering their structure and characteristics and achieving improved biocompatibility [32,33,34]. Nevertheless, the application of PANI becomes restricted due to its low processability and insolubility in the most common solvents. To overcome this limitation, a modified breath figure (BF) method combined with interfacial polymerization to functionalize PANI on a micro-structured film surface was reported [35,36]. The BF method is a simple and convenient method to fabricate highly ordered honeycomb-patterned (HCP) porous films with specific surface topography and chemical distribution, first reported by Widawski et al. [37]. Many researchers have reported the antibacterial and antifouling activity of HCP films by incorporating antibacterial and antibiofilm moieties such as TiO_2_ [24], silver NPs [38,39], silicone oil [40], etc. However, the antibacterial activity of PANI-coated HCP films has not been reported yet.

In the present report, we studied changes in the antibacterial activity of functionalized PANI with different surface topographies and dopant used. We fabricated three kinds of PANI-coated film: flat PANI, HCP PANI, and HCP sulfonated PANI. The HCP porous PANI films were fabricated using the modified BF method via a 2-step process. For the fabrication of flat and HCP functionalized PANI films, PCL was used as a template and benzoyl peroxide (BPO) was used as an oxidant. Later, the functionalization of PANI was achieved by immersing the PCL films in an aqueous solution of aniline hydrochloride solution for the interfacial polymerization reaction. Additionally, H_2_SO_4_ was added to the aniline hydrochloride solution during the polymerization to obtain sulfonated HCP PANI films for zwitterionic surface modification. These films were then checked for antibacterial properties using the disk diffusion and microdilution method. The antibiofilm activity was also checked by crystal violet assay.

## 2. Materials and Methods

### 2.1. Materials

Poly (ε-caprolactone) (PCL) (Mn = 70,000–90,000 g mol^−1^), aniline hydrochloride (C_6_H_5_NH_2_·HCl, 99%), benzoyl peroxide (BPO, C_14_H_10_O_4_, 95%), 1-methyl-2-pyrrolidinone (NMP), and agar were procured from Sigma-Aldrich (South Korea). Tetrahydrofuran (THF) and sulfuric acid (H_2_SO_4_) were purchased from SK Chemicals (South Korea). Crystal Violet was purchased from Samchun Chemicals (South Korea). Luria–Bertani (LB) broth was purchased from BD Difco (UK). All of the chemicals obtained were used as received without further purification. *Escherichia coli* (*E. coli*) and *Staphylococcus aureus* (*S. aureus*) were obtained from the Korean Collection for Type Culture and the Korean Culture Center of Microorganisms, respectively.

### 2.2. Fabrication of Films

We fabricated flat and HCP porous PANI films for the topographical comparison and HCP porous PANI and HCP porous sulfonated PANI films for the comparison of the dopant acids.

#### 2.2.1. Preparation of f-PANI Film

For the preparation of the f-PANI film, at first, a flat PCL film was fabricated. In this study, we used 5 wt. % of BPO and prepared a polymer solution using THF. The polymer solution with BPO was poured into a glass Petri dish and air-dried. After the evaporation of the solvent, i.e., THF, a translucent flat PCL film was obtained. Later, this film was dipped in 0.2M aqueous aniline hydrochloride solution, and the BPO present in the PCL film initiated the interfacial polymerization, which occurred at the interface (surface) of the PCL film. This chemical oxidation reaction was maintained at 0–4 °C for 1 h with constant stirring. After the completion of the functionalization, the film was taken out and washed with distilled water to remove loosely bound monomers of PANI, followed by air-drying it. After the polymerization, a green colored film was obtained and will be further denoted as f-PANI.

#### 2.2.2. Preparation of HCP Porous PANI Film

The PCL films were fabricated via the BF method previously described by our group [41]. The same concentration of BPO as that for the f-PANI film was used, and the prepared polymer solution was cast inside an in-house BF chamber, maintained at 70–80% relative humidity. After complete drying, an opaque PCL HCP film was obtained, further addressed as PCL film. For the preparation of HCP porous PANI films, the same procedure as above was used with the HCP PCL film obtained after the BF method. The parameters were kept similar for the interfacial polymerization of the HCP PCL film. After the completion of functionalization, the films were taken out and washed, followed by air drying. During polymerization, the sequence of colorations of the film changed from white to light blue, then blue-green, and finally dark green. The dark green color is the characteristic color of emeraldine salt (ES), which is a conductive form of PANI. The fabricated film will be further denoted as HCP-PANI.

#### 2.2.3. Preparation of HCP Porous Sulfonated PANI Films

**Figure 1:**
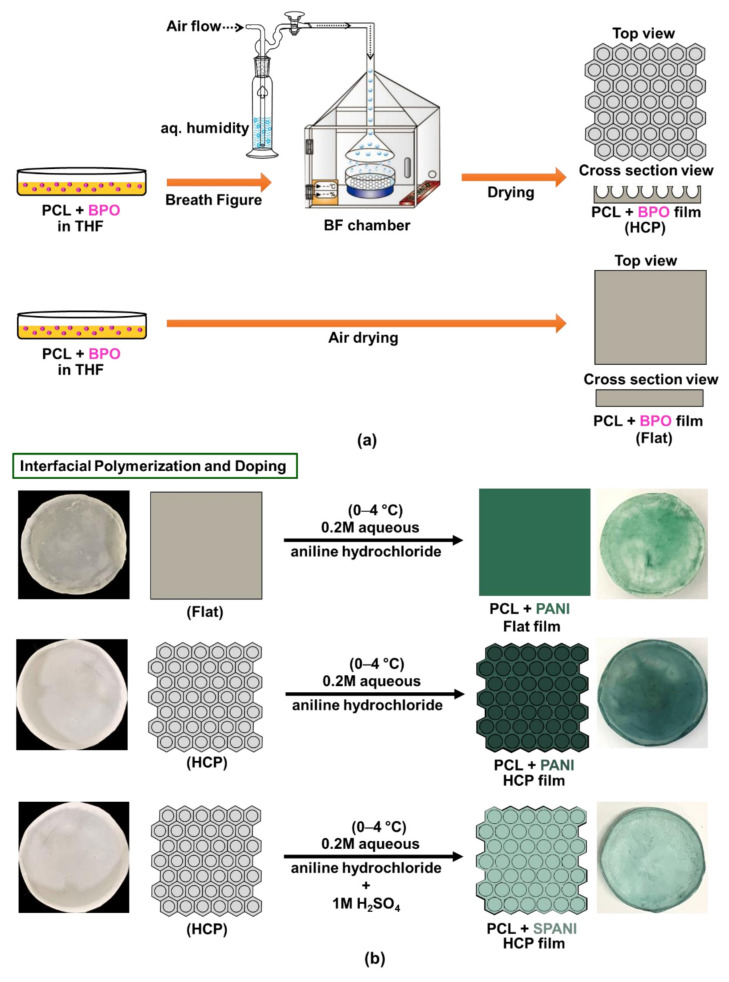
The overall scheme of PANI functionalization. (**a**) Fabrication of HCP (**top**) and flat (**bottom**) PCL films. (**b**) Interfacial polymerization of PANI at the surface of flat (**top**), HCP (**middle**), and sulfonated HCP (**bottom**) films with their corresponding photo images.

For the preparation of sulfonated PANI films, 1M H_2_SO_4_ was added to the aniline hydrochloride solution as a dopant to produce a zwitterionic surface. The HCP PCL film was dipped into this solution and maintained at 0–4 °C for 1 h under stirring. After functionalization, the film was taken out, washed, and air-dried. This sulfonated-PANI functionalized film will be further denoted as HCP-SPANI. Doping with H_2_SO_4_ resulted in a light green coloration of the HCP-SPANI films. A detailed experimental scheme for the formation of f-PANI, HCP-PANI, and HCP-SPANI films with their photographic images is shown in Figure 1.

### 2.3. Characterization

The morphologies of the fabricated films (PCL, f-PANI, HCP-PANI, and HCP-SPANI) were characterized by scanning electron microscopy (SEM; CX-100 model, COXEM). The composition distribution of film surface was determined by energy dissipation X-ray spectroscopy (EDX) analysis using FESEM (JSM-6700F, JEOL, Tokyo, Japan) equipped with electron backscatter diffraction (Pegasus with Hikari). The analysis was performed by covering a relatively large sample area and using a high voltage (20 keV) maximized resolution. Fourier transform infrared spectroscopy (FT-IR) analysis was performed by using a Varian 1000 FT-IR spectrometer (Scimitar series, Varian Inc., Palo Alto, CA, USA) at a resolution of 4 cm^−1^ and with an average of 42 scans in the range of 400–4000 cm^−1^. The UV–vis spectra of the films were obtained using a UV-visible spectrophotometer (Waters-996). Thermogravimetric analyses (TGA) were performed with a thermal analyzer (TGA Q50, TA Instruments, New Castle, DE, USA) at a ramp rate of 5 °C min^−1^, from ambient temperature to 800 °C under argon purge with a flow rate of 40 mL min^−1^. The surface wettability was characterized by using contact angle measurement via contact angle analyzer (Model no. CAM 100, KSV Co., Busan, Korea). The conductivity measurements were obtained by four-point probe technique using Keithley 6220 constant current source and Keithley 2182A digital electrometer. The contact angle and conductivity were measured at least three times on different locations of each film, and the average value was plotted. The absorbance was measured at 570 nm and 600 nm by the microplate reader (BioTek PowerWave XS, Santa Clara, CA, USA).

### 2.4. Assessment of Antibacterial and Antibiofilm Activity of Fabricated Films

The antibacterial property of the fabricated films was investigated by the disk diffusion and broth dilution methods. [42,43] The antibiofilm activity of the films was investigated by the crystal violet assay [13,44].

#### 2.4.1. Disk Diffusion Assay

The cultures of *E. coli* (Gram-negative) and *S. aureus* (Gram-positive) were cultured from single colonies that were incubated overnight at 37 °C in LB broth (BD Difco, England, UK). The turbidity of the cultured bacterial suspensions was measured in terms of optical density at 600 nm (OD_600_) by using a spectrophotometer until the 0.50 McFarland standard was achieved. Then, 100 μL of each strain of *E. coli* and *S. aureus* were used and swabbed uniformly onto the individual LB agar plates using sterile swabs [42,43]. The fabricated films, namely PCL, f-PANI, HCP-PANI, and HCP-SPANI, were cut into discs (~5 mm in diameter) and placed onto the LB plates, previously inoculated with the test bacteria, and then incubated at 37 °C for 18 h. Here, the PCL film was used as a control. After incubation, the diameters of the inhibition zones were measured on a millimeter scale using ImageJ software.

#### 2.4.2. Broth Microdilution Method

For the microdilution method, sterile microtubes (1.5 mL) were loaded with fabricated films that had been cut into 0.5 cm × 0.5 cm pieces. Next, the overnight culture was diluted at 1:100 to prepare the culture and was used for the experiment. One milliliter of the prepared bacterial culture was pipetted onto each substrate, placed aseptically inside a microtube [42,43]. The lid of the microtube was closed to prevent the sample from drying out. The PCL, f-PANI, HCP-PANI, and HCP-SPANI films were incubated with the prepared culture at 37 °C with shaking at 150 rpm. Here, the PCL film was used as a control. After 24 h, the absorbance (Abs.) of all samples was taken at OD_600_, and the corresponding bacterial growth graph was plotted. All tests were performed in triplicate. The percentage of cell growth can be calculated by Equation (1).

(1) $$\mathrm{Cell}\;\mathrm{growth}\;\%=\frac{Initial\;Abs.-Final\;Abs.}{Initial\;Abs.}\times 100$$

#### 2.4.3. Antibiofilm Activity

To investigate the antibiofilm activities of the fabricated films against *E. coli* and *S. aureus,* the static biofilm formation was assayed in 24-well polystyrene plates [13,44]. In brief, cells in LB at initial turbidity of 0.05 at 600 nm (OD_600_) were cultured with all of the fabricated films (PCL, f-PANI, HCP-PANI, and HCP-SPANI) for 24 h without shaking at 37 °C. The PCL film was used as a control. To quantify total biofilm formation, biofilms in 24-well plates were stained with 0.1% crystal violet for 20 min, dissolved in 95% ethanol, and then absorbance was measured at 570 nm (OD_570_). Results are the averages of measurements taken from at least three replicates. Further, the biofilms were imaged using SEM. The percentage of biofilm formation can be calculated by Equation (2).

(2) $$\mathrm{Biofilm}\;\mathrm{formation}\;\%=\frac{Initial\;Abs.-Final\;Abs.}{Initial\;Abs.}\times 100$$

### 2.5. Statistical Analysis

To determine the statistical significance of the differences between the means of different experimental groups in terms of their antibacterial activity and bacterial cell attachment, a one-way analysis of variance (ANOVA) test was performed. Data were obtained at least in triplicate (*n* = 3), averaged, and expressed as mean ± standard deviation (SD). A *p*-value of less than 0.05 was considered statistically significant.

## 3. Results and Discussions

### 3.1. Characterization

**Figure 2:**
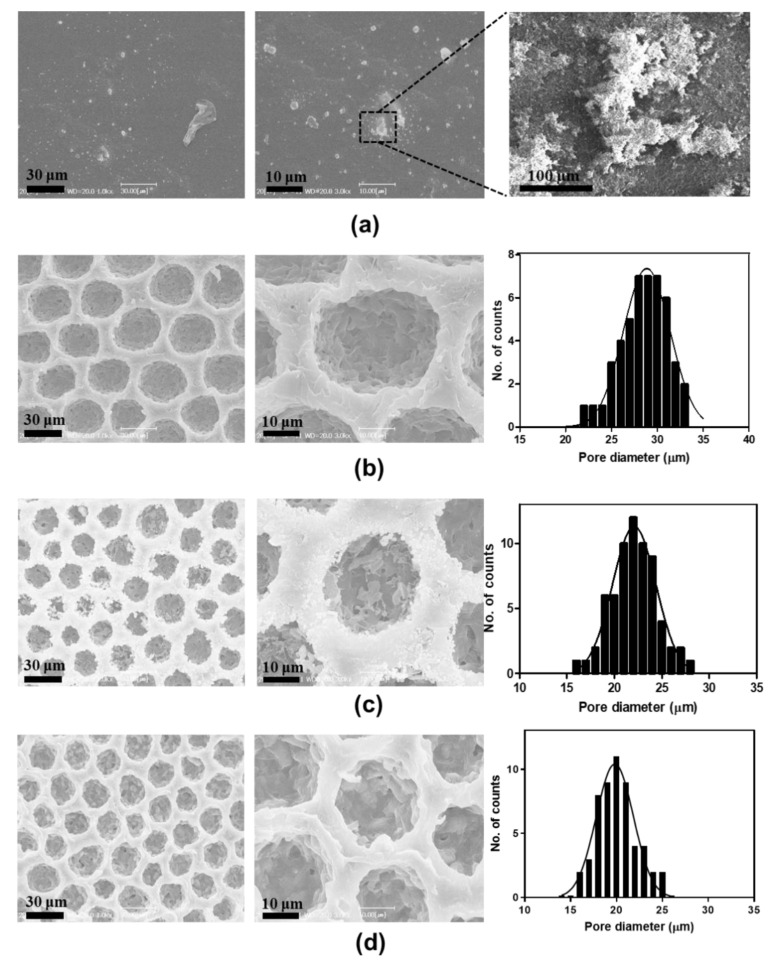
SEM images of (**a**) f-PANI, (**b**) PCL, (**c**) HCP-PANI, and (**d**) HCP-SPANI films. There was no significant difference in the HCP pattern of the HCP films, but a difference in morphology of PANI was observed functionalized on different films, such as flat, HCP, and sulfonated HCP.

Primarily we compared the morphology of functionalized PANI films on differences in topography and chemical composition. Figure 2 shows the SEM images of f-PANI, PCL, HCP-PANI, and HCP-SPANI films at low (10 μm) and high (3 μm) magnifications. The SEM image of f-PANI film shows a flat surface with randomly distributed aggregates (Figure 2a). These aggregates might be of PANI, as PANI tends to randomly aggregate in the aqueous phase due to the formation of hydrophobic nucleates, followed by the growth of PANI chains from the self-assembled nucleates [45]. Figure 2b shows the SEM images of PCL film with HCP morphology. The HCP was regular with an average pore diameter of 29 ± 2 μm. Figure 2c shows the SEM images of HCP-PANI films having regular HCP pores with granular structures with nanofibers present all over the film surface. These granular protrusions might be of PANI, as that is the most typical morphology of PANI produced by the polymerization of aniline in strong acidic conditions [45]. Additionally, doping with HCl facilitates the growth of PANI fiber structures [46]. The average pore diameter of pores was 22 ± 3 μm, shown in the corresponding pore-distribution histogram. Figure 2d shows uniform HCP porous surface morphology of HCP-SPANI films with a mean pore diameter of 20 ± 3 μm. The HCP-SPANI film indicates layer-by-layer stacked chunks, which might be of PANI, as doping with H_2_SO_4_ prohibits the growth of PANI fibers due to the strong interaction between H_2_SO_4_ and PANI during the polymerization, as reported by Ingle et al. [46]. The average pore size of HCP-PANI and HCP-SPANI films was lower than that of PCL film, which might be due to the polymerization of PANI inside and outside the pores, leading to lower pore size. A substantial difference was observed in the morphology of PANI in the different topographies as well as in the presence of two different acids. Therefore, these results indicate that under similar reaction conditions such as reaction time, aniline hydrochloride concentration, and temperature, different morphologies of PANI can be obtained by using different topographies and dopant acids.

**Figure 3:**
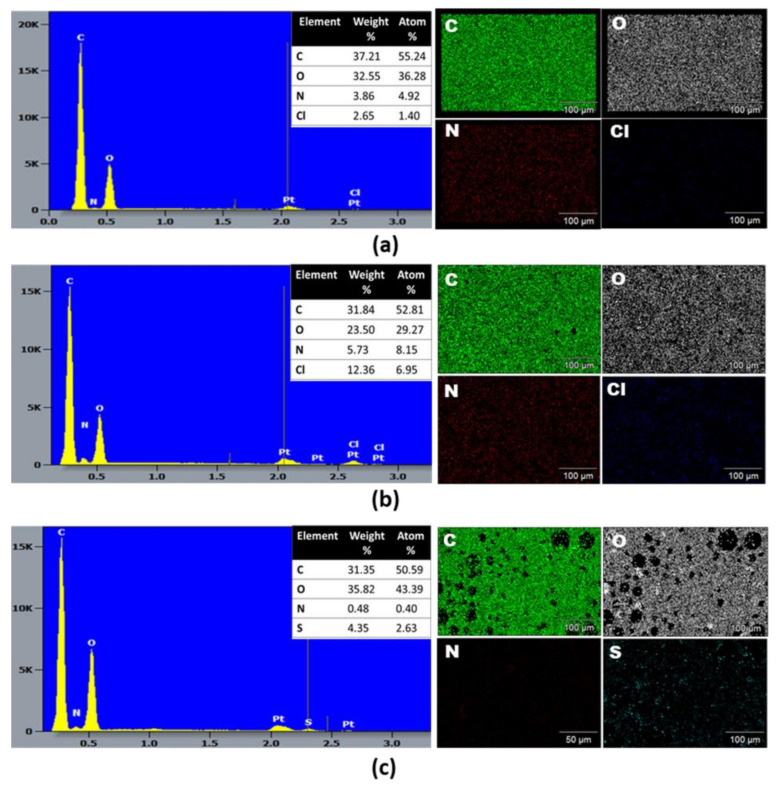
SEM-EDX and qualitative-quantitative elemental mapping analysis of (**a**) f-PANI, (**b**) HCP-PANI, and (**c**) HCP-SPANI films. All three films showed the presence of carbon (C), oxygen (O) and nitrogen (N), and additionally chlorine (Cl) for the f-PANI and HCP-PANI films, while the presence of sulfur (S) was observed in HCP-SPANI, confirming the doping of HCl and H_2_SO_4_, respectively.

The EDX spectra and qualitative-quantitative elemental mapping were analyzed to determine the overall chemical composition and the distribution of the chemical elements of the f-PANI, HCP-PANI, and HCP-SPANI films, shown in Figure 3. Strong peaks of carbon (C) and oxygen (O) were observed in all of the films as the main components, which may be attributed to the C and O present in the PCL backbone [47]. Additionally, the f-PANI and HCP-PANI films showed nitrogen (N) and chlorine (Cl), which indicated the surface functionalization of both films with PANI [41]. The presence of Cl validated that PANI was present in the ES form, as HCl was present as a dopant, shown in Figure 3a,b. The most interesting aspect regarding interfacial polymerization in the corresponding tables showed the elemental weight and atom %, indicating an increase in the weight and atom % of Cl from 2.65% and 1.46% in f-PANI to 12.36% and 6.95% in HCP-PANI films. The quantitative analysis suggested that PANI functionalization was more extensive on the HCP patterned film than on the flat film, even though all of the experimental conditions were similar for the fabrication of both films. This might be because HCP films have higher surface area than flat films; therefore, more functionalization of PANI was obtained. [7,48] By contrast, sulfur (S) was detected in the HCP-SPANI films, which confirmed the sulfonation of the surface by H_2_SO_4_ used as a dopant, shown in Figure 3c [49]. It should also be noted that the oxygen content was highest (43.39%) in the HCP-SPANI film, further confirming the presence of the −SO_3_H group, which was attributed to the zwitterionic polymer film surface.

**Figure 4:**
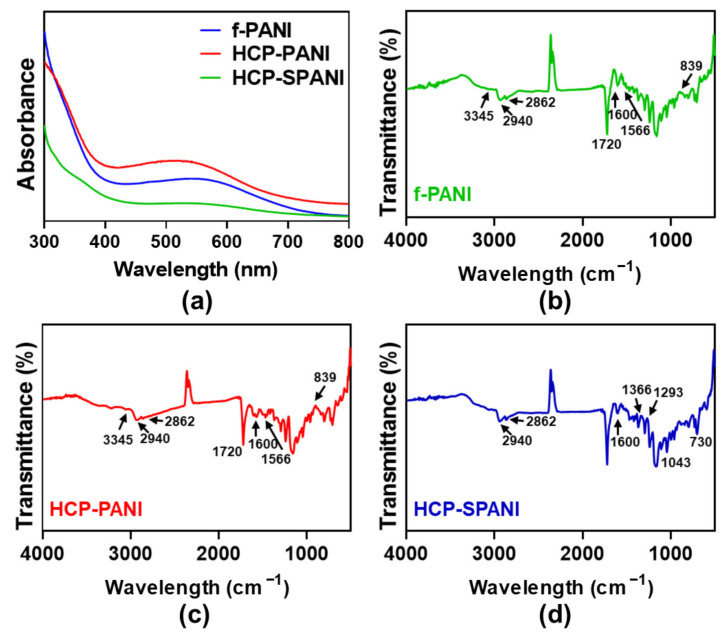
Characterization of fabricated films. (**a**) UV-Vis and (**b**–**d**) FT-IR spectra of the f-PANI, HCP-PANI, and HCP-SPANI films. The data indicate the functionalization of PANI (ES) and sulfonated PANI on the fabricated films.

Figure 4 shows the chemical and physical characteristics of the fabricated films. Figure 4a shows the UV-Vis spectrum recorded for f-PANI, HCP-PANI, and HCP-SPANI films in the range of 300–800 nm. The f-PANI, HCP-PANI, and HCP-SPANI films were dissolved in NMP. The spectra of f-PANI and HCP-PANI presented broad bands with the maximum at 510 nm, which represents π–π* electronic transitions related to the benzenoid form of PANI and polaron→π* electronic transitions related to the quinoid rings of PANI, respectively [35,36]. The absorption spectra of HCP-SPANI showed peaks around 500–600 nm for sulfonated PANI assigned to π→polaron electronic transitions [50,51]. Figure 4b–d shows the FT-IR spectra of the fabricated films. In the FT-IR spectra of f-PANI and HCP-PANI, the peak formed at a frequency of 839 cm^− 1^ represents the N–H flexural bonds of the functional groups participating in hydrogen bonds. The presence of benzenoid bonds at a frequency of 1566 cm^− 1^ indicated that PANI was used in the form of ES. The presence of the peak at a frequency of 3345 cm^− 1^ represented the symmetric and asymmetric tensile bonds of the N–H aromatic ring in PANI, as shown in Figure 4b,c [52]. The most important characteristic bands for PANI were the band observed at a wavelength of 1600 cm^−1^ and the band at 1720 cm^−1^, which represented the benzenoid and quinoid rings, respectively [53]. The main peaks at 1600 cm^− 1^ and 1366 cm^−1^ in the spectrum of HCP-SPANI shown in Figure 4d corresponded to quinone- and benzene-ring stretching deformations, respectively. The absorption band at 1293 cm^−1^ corresponded to p-electron delocalization induced in the polymer by protonation. The peak at 1293–1365 cm^−1^ could be related to the aromatic amine and sulfate compounds resulting from the dopant acid. Two additional peaks at 1043 cm^−1^ and 730 cm^−1^ were observed from the spectrum of HCP-SPANI and were attributed to the symmetric stretching vibration and stretching vibration of the S−O bond, respectively. [20] In all of the samples, the main characteristic peaks of PCL were observed at ~1721 cm^− 1^ (carbonyl stretch) and ~2940 cm^− 1^ (CH_2_ asymmetric stretch) [54], followed by the other peaks including symmetric CH_2_ stretching at ~2862 cm^−1^, stretching of C–O and C–C at ~1293 cm^−1^, asymmetric C–O–C at ~1238 cm^−1^, and symmetric C–O–C around 1170 cm^−1^ that were observed in all PCL-containing films.

**Figure 5:**
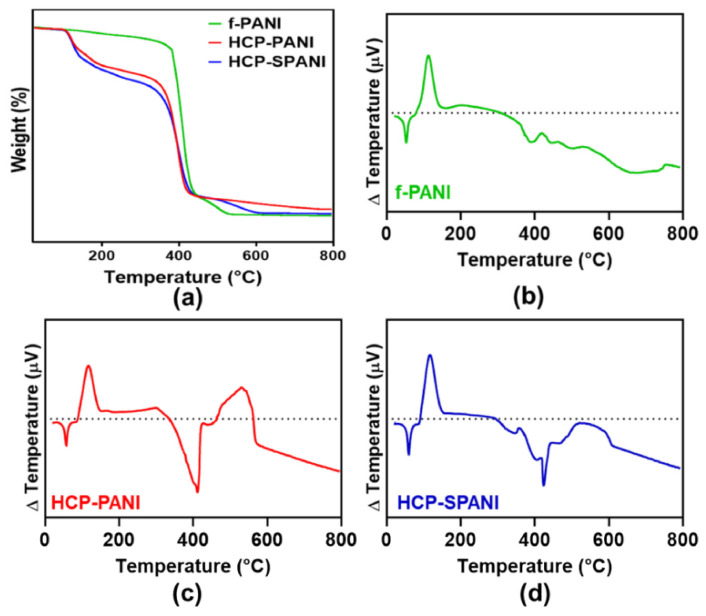
Characterization of fabricated films. (**a**) TGA and (**b**–**d**) DTA analysis of the f-PANI, HCP-PANI, and HCP-SPANI films.

Figure 5a represents the thermogravimetric thermograms of f-PANI, HCP-PANI, and SPANI films. The thermogram of f-PANI showed a three-step degradation. In the first degradation step, around 8.4% weight loss was observed from 287.39 °C to 330.72 °C. The major weight loss, of about 83.85%, was observed in the second degradation step between 384.96 °C and 408.98 °C. In the final degradation step, around 11.8% of weight loss was observed between 442.85 °C and 724.74 °C. The degradation of f-PANI between 150 to 400 °C was similar to the PCL degradation curve [55]. This might be due to the lower functionalization of PANI on the flat film; therefore, a characteristic PCL curve was obtained in that range. The thermogram of HCP-PANI showed a three-step degradation behavior. In the first degradation step, weight loss of 20.26% was observed from 112.81 °C to 143.84 °C. In the second degradation step, weight loss of 72.15% was observed between 370.06–450.18 °C. In the third degradation step, 6.04% weight loss was observed between 550.72–660.78 °C. The thermogram of HCP-SPANI showed a three-step degradation behavior. In the first degradation step, 16.41% weight loss was observed from 104.59 °C to 128.11 °C. In the second degradation step, 78.95% weight loss was observed between 130.35–414.02 °C. In the final degradation step, the weight loss was around 8.5% in the temperature range of 503.79 °C to 583.82 °C. The degradation below 150 °C in f-PANI, HCP-PANI, and HCP-SPANI films might be due to the physically adsorbed water molecules and the degradation of BPO. The weight loss between 100 and 300 °C might be because of the decomposition of dopants in the HCP-PANI and HCP-SPANI films [56]. The degradation above 400 °C was mostly due to the degradation of the PANI backbone, as PANI is a relatively more stable polymer than PCL [41]. Figure 4b–d shows the DTA of f-PANI, HCP-PANI, and HCP-SPANI films. The DTA curves show endothermic peaks around 61 °C for f-PANI, HCP-PANI, and HCP-SPANI films because of the presence of PCL in the films, while an exothermic peak was observed at 115 °C, which could be attributed to the degradation of dopants. HCP-SPANI showed sharp endothermic peaks at 410 °C and a broad exothermic peak around 580–610 °C. An exothermic peak at 450–500 °C was also observed in all films and is a characteristic peak for PCL [57]. Overall, these results indicate the successful doping of the PANI films with different acids.

**Figure 6:**
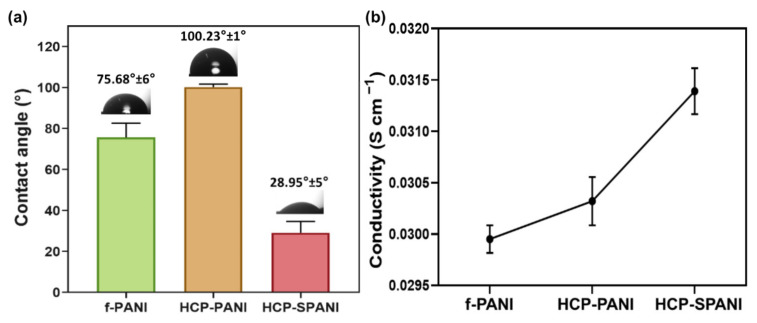
(**a**) Water contact angle and (**b**) conductivity of f-PANI, HCP-PANI, and HCP-SPANI films.

Figure 6a exhibits the wetting ability of f-PANI, HCP-PANI, and HCP-SPANI films. The water contact angle of the f-PANI film was 75.68° ± 6 °. However, the contact angle of HCP-PANI showed a higher value of 100.23° ± 1°, which might be due to the HCP surface. The patterned surfaces play an important role in the wetting behavior, and the HCP porous structures tend to show higher contact angle values than flat films, which may be explained by the Wenzel and Cassie theories [58]. A decreased contact angle value of 28.95° ± 5° was observed for the HCP-SPANI film, indicating that sulfonation was able to improve the wetting ability of HCP-SPANI films [20]. Doping of PANI with additional acids makes it more hydrophilic because of the positive charge group of N^+^ belonging to the quinoid unit along the polymer backbone [59]. Figure 6b shows the conductivity values for f-PANI, HCP-PANI, and HCP-SPANI films. The conductivities of the f-PANI and HCP-PANI films were 2.99 × 10^−2^ and 3.05 × 10^−2^ S cm^−1^, respectively. This difference in conductivity might be due to the HCP structure of the film, which increases the surface area and, therefore, more PANI was functionalized on the surface compared to f-PANI film, as observed in Figure 2 and Figure 3. Doping of PANI with strong acids further increased the conductivity of the HCP-SPANI films to 3.15 × 10^−2^ S cm^−1^. It is well known that PANI shows good conductivity in acidic media, as protons participated in the redox process of PANI. The doping of PANI with strong acid inserts sulfonic acid groups in the polymer chain, which could change the microenvironment of the nitrogen atoms and shift the local pH [60]. Therefore, the conductivity was enhanced by the topographical as well as surface modification by HCP and sulfonation.

**Figure 7:**
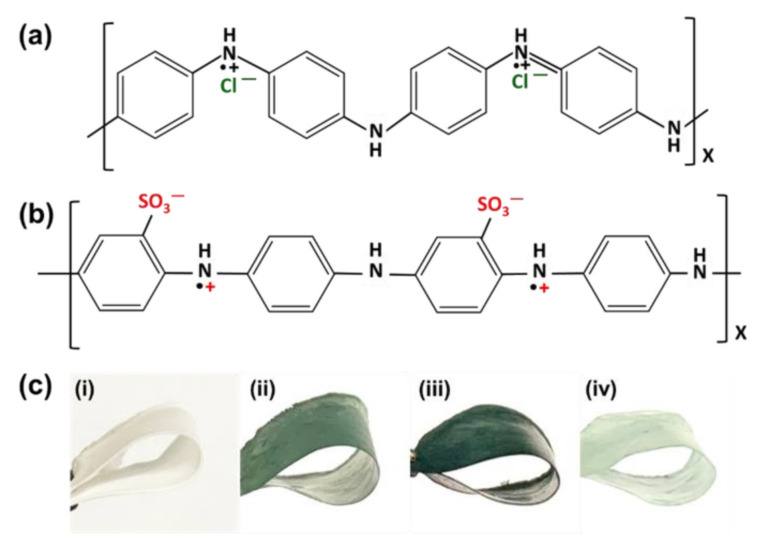
Chemical structure of (**a**) PANI ES in HCl, and (**b**) PANI ES in H_2_SO_4_. Doping of positive and negative charges on the backbone of the PANI chain for the fabrication of a zwitterionic surface. (**c**) Flexibility of the prepared films (i) PCL, (ii) f-PANI, (iii) HCP-PANI, and (iv) HCP-SPANI.

It is quite evident from the results that the PANI was efficiently doped with H_2_SO_4_ in a single-step process. The chemical structure of PANI ES in the presence of HCl is presented in Figure 7a. After doping, the polarons are delocalized, yielding a green conducting ES state [53]. In addition, in the case of self-doping, both positive charge and negative charge are along the chain bones of sulfonated PANI after sulfonation reaction with H_2_SO_4_ acid, as shown in Figure 7b. A modification of the zwitterionic surface on the HCP-PANI surface is theoretically possible based on the resistance to strong acid solutions and the doped properties of PANI, exhibiting the characteristics of zwitterionic polymer, which is considered to be one of the best types of antifouling materials [61]. The PCL, as the main backbone of the polymer, provided flexibility to the prepared f-PANI, HCP-PANI, and HCP-SPANI films, and even after polymerization with PANI, the films remained quite flexible, as shown in Figure 7c. Thus, using PCL as a template offers flexibility and biocompatibility, and functionalizing PANI on it provides doping-dedoping, conductivity, and antibacterial properties to the fabricated films.

### 3.2. Antimicrobial Assessment

**Figure 8:**
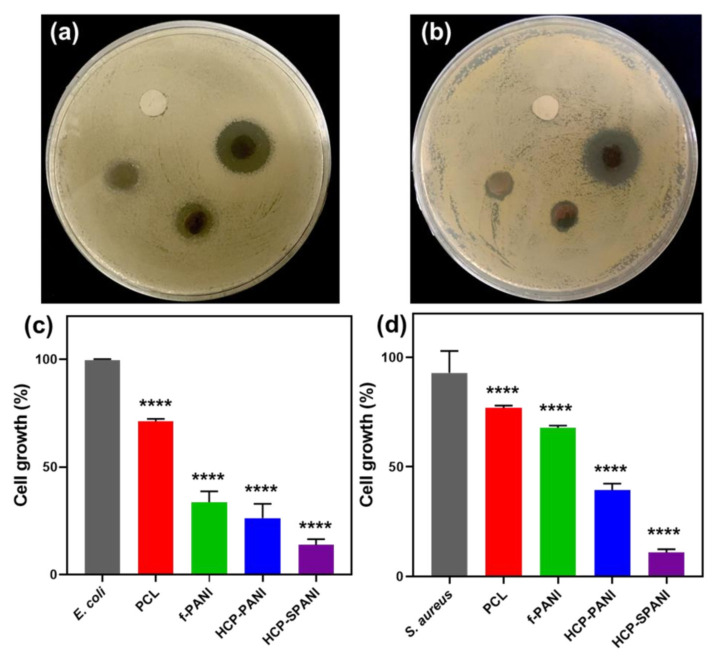
Antibacterial activity of the fabricated films. Disk diffusion assay of fabricated films against (**a**) *E. coli* and (**b**) *S. aureus*, and the bacterial growth % of (**c**) *E. coli* and (**d**) *S. aureus* on PCL, f-PANI, HCP-PANI, and HCP-SPANI films after 24 h measured at OD _(600 nm)_. The data are averages of three independent experiments performed in triplicate, **** *p* < 0.0001.

To study the antibacterial properties of the surfaces, we investigated the cell growth and biofilm formation of *E. coli* and *S. aureus* on the flat and HCP films. Primarily, a disc-diffusion assay followed by broth micro dilutions analysis was performed to evaluate the antibacterial activity [42]. Here, PCL film was used as a control. Figure 8 shows the antibacterial activity of the fabricated films against *E. coli* and *S. aureus*. Figure 8a,b shows the disc-diffusion assay, in which it is observed that PCL does not show antibacterial activity against either strain. The f-PANI, HCP-PANI, and HCP-SPANI films showed a zone of inhibition against both strains. The f-PANI and HCP-PANI films showed a zone of inhibition of about 1.82 ± 0.65 mm and 2.71 ± 0.14 mm against *E. coli*, and 1.54 ± 0.01 mm and 1.74 ± 0.09 mm against *S. aureus*, respectively. The maximum zone of inhibition was detected in HCP-SPANI films for both strains, which were 6.44 ± 0.27 mm and 6.42 ± 0.28 mm against *E. coli* and *S. aureus*, respectively, as shown in Table 1. The microdilution assay also showed good antibacterial activity against both of the tested strains. We observed that the f-PANI and HCP-PANI films inhibited cell growth of *E. coli* by over 69.79% and 78.27%, and of *S. aureus* by 32.73% and 62.65%, respectively. The HCP-SPANI film showed maximum inhibition of around 88% and 87.97% against *E. coli* and *S. aureus*, respectively, as shown in Figure 8c,d. In addition, the one-way ANOVA test showed statistically significant results against both strains (*p* < 0.0001). The results are consistent with previously reported studies showing that PANI is antibacterial [52,62,63]. Therefore, the HCP-PANI films demonstrated enhanced antibacterial activity, and more than that of f-PANI film and the doped PANI film, i.e., HCP-SPANI showed more enhanced activity than the HCP-PANI films in the same concentration. The conducting polymers can cause cell death by disrupting microbial cell walls and membranes via electrostatic contact [64]. Similarly, doped PANI films may possess other potent antimicrobial compounds, in particular cationic antimicrobial peptides that interact with anionic bacterial membranes and compromise membrane integrity and cell division, leading to cell lysis and death and, therefore, showing good antibacterial activity [62].

**Figure 9:**
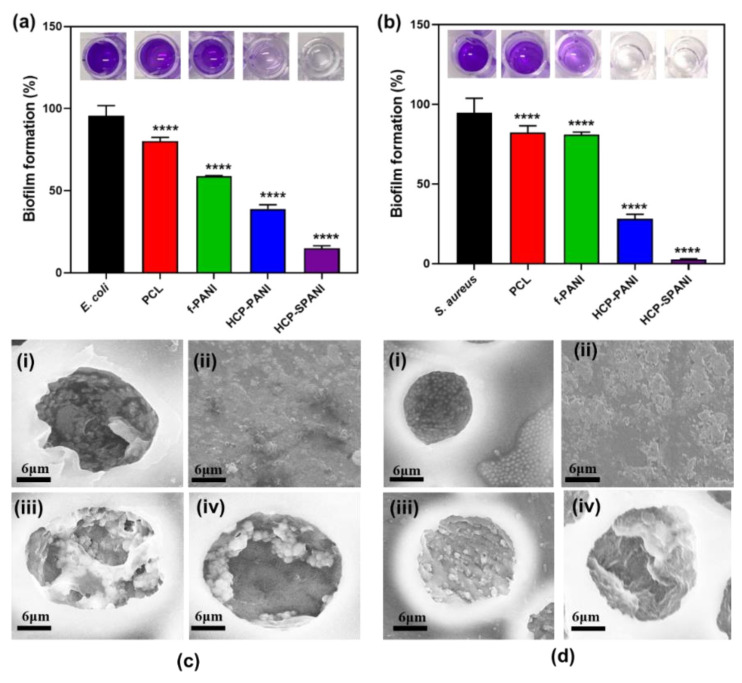
Antibiofilm activity of the fabricated films against (**a**) *E. coli* and (**b**) *S. aureus* after 24 h of biofilm growth measured at OD _(570 nm)_. The data are averages of three independent experiments performed in triplicate, **** *p* < 0.0001. SEM images of (**i**) PCL, (**ii**) f-PANI, (**iii**) HCP-PANI, and (**iv**) HCP-SPANI films after 24 h of biofilm assay against (**c**) *E. coli* and (**d**) *S. aureus*.

After assessment of the antibacterial activity, the antibiofilm activity of the fabricated films was evaluated by crystal violet staining [13,44]. Here, PCL film was used as a control. Figure 9a depicts blue color microwells for control samples of *E. coli*, PCL, and f-PANI films, while transparent microwells for *E. coli* against HCP-PANI and HCP-SPANI films indicated the antibiofilm activity of these films with a decrease in absorbance (OD_570_). For the antibiofilm activity of the films against *S. aureus,* similar results were observed, as shown in Figure 9b. The f-PANI and HCP-PANI films inhibited *E. coli* biofilm formation by around 41.62% and 63% and that of *S. aureus* by 17.81% and 69.83%, respectively. The HCP-SPANI films inhibited *E. coli* biofilm formation by around 83.88% and that of *S. aureus* by 96.57%, respectively. The one-way ANOVA test showed statistically significant results against both strains (*p* < 0.0001) for the biofilm assay. Cumulatively, all of the results confirmed that the fabricated films had antibacterial and antibiofilm activity against both strains, with HCP-SPANI showing the maximum inhibition. The improved hydrophilicity provided the basis for the antifouling properties of modified HCP-SPANI films, as shown in Figure 6a. Moreover, the antibiofilm activity of the fabricated films might be due to the presence of zwitterionic groups, which show remarkable antifouling activities, and high hydrophilicity, which might be due to the presence of minimized dipoles and balanced charges [65,66]. The antibiofilm activity of these polymers is attributed to the hydrogen bonding ability and static-induced hydration layer formation. The hydration layer can lead to a strong repulsive force and, therefore, show high resistance to cell adhesion, biofilm formation, and nonspecific protein adsorption [67]. These results illustrate that the doped films were versatile toward resisting the attachment of bacteria and the subsequent formation of biofilms by both Gram-negative (*E. coli*) and Gram-positive (*S. aureus*) bacteria.

Figure 9c,d shows the typical SEM images of all the fabricated films after 24 h of biofilm assay. Large aggregates of *E. coli* were observed inside the pores of the PCL films, as shown in Figure 9c(i). Similar aggregation was observed for *S. aureus*, which was present in a matrix inside the pores as well as on the interpore surfaces of the PCL films, as shown in Figure 9d(i). The f-PANI showed aggregates of bacteria and biofilm formation for both strains, as shown in Figure 9c(ii),d(ii). In the case of HCP-PANI films, slight biofilm formations and cell aggregates of *E. coli* were observed (Figure 9c(iii)), while no biofilm and few cells of *S. aureus* were observed (Figure 9d(iii)). Biofilm formation on the HCP-SPANI film was disrupted, and few to no cell aggregates of *E. coli* were found inside the pores of the HCP-SPANI films, as shown in Figure 9c(iv). In the case of *S. aureus*, no biofilm formation and no cell aggregates were observed in the HCP-SPANI film, as shown in Figure 9d(iv). Additionally, the bacterial cells tended to migrate inside the pores of the HCP films, leaving the interpore surfaces free from cells [38]. The SEM images validate the results of the crystal violet biofilm assay shown in Figure 9a,b.

## 4. Conclusions

In summary, the antibacterial activity of PANI functionalized on different topographies was studied together with the dopant effect by sulfonation. The modified BF method was used to synthesize the HCP films followed by interfacial polymerization for the surface coating of PANI using different dopant acids. For the topographical comparison, PANI was coated on the flat PCL film. SEM images revealed a regular HCP surface with a decrease in pore size of the HCP-PANI and HCP-SPANI films due to the coating of PANI on the surfaces. Additionally, different morphologies of PANI were observed with different topographies and chemical modifications. When measured by contact angle as 75.68° ± 6°, the wettability of f-PANI was higher than that of HCP-PANI film, with a contact angle corresponding to 100.23° ± 1° that was further increased by the sulfonation of HCP-PANI film (28.95° ± 5°). Briefly, the antibacterial activity of the films was as follows: HCP-SPANI > HCP-PANI > f-PANI. The difference in antibacterial activity between the flat and HCP PANI films might be due to more functionalization of PANI on HCP film because of the relatively high surface area. The sulfonated HCP-PANI film showed the highest antibacterial and antibiofilm activity, which might be due to the modified zwitterionic surface. Therefore, a hydrophilic surface was achieved, which inhibited bacterial attachment and showed enhanced antibiofilm activity.

## Figures and Tables

**Table 1:** The zone of inhibition of the fabricated films against *E. coli* and *S. aureus*.

Strain	Zone of Inhibition (mm)			
	PCL	f-PANI	HCP-PANI	HCP-SPANI
*E. coli*	0	1.82 ± 0.65	2.71 ± 0.14	6.44 ± 0.27
*S. aureus*	0	1.54 ± 0.01	1.74 ± 0.09	6.42 ± 0.28

## Data Availability

Not applicable.
